# Association of ABO Blood Group Phenotype and Allele Frequency with Chikungunya Fever

**DOI:** 10.1155/2015/543027

**Published:** 2015-04-21

**Authors:** Pairaya Rujirojindakul, Virasakdi Chongsuvivatwong, Pornprot Limprasert

**Affiliations:** ^1^Department of Pathology, Faculty of Medicine, Prince of Songkla University, Hat Yai, Songkhla 90110, Thailand; ^2^Epidemiology Unit, Faculty of Medicine, Prince of Songkla University, Hat Yai, Songkhla 90110, Thailand

## Abstract

*Background*. The objective of this study was to investigate the association of the ABO blood group phenotype and allele frequency with CHIK fever. *Methods*. A rural community survey in Southern Thailand was conducted in August and September 2010. A total of 506 villagers were enrolled. Cases were defined as individuals having anti-CHIK IgG by hemagglutination ≥1 : 10. *Results*. There were 314 cases (62.1%) with CHIK seropositivity. Females were less likely to have positive anti-CHIK IgG with odds ratio (OR) (95% CI) of 0.63 (0.43, 0.93). All samples tested were Rh positive. Distribution of CHIK seropositivity versus seronegativity (*P* value) in A, B, AB, and O blood groups was 80 versus 46 (0.003), 80 versus 48 (0.005), 24 versus 20 (0.55), and 130 versus 78 (<0.001), respectively. However, chi-square test between ABO and CHIK infection showed no statistical significance (*P* = 0.76). Comparison of the ABO blood group allele frequency between CHIK seropositivity and seronegativity was not statistically significant. *Conclusion*. This finding demonstrated no association of the ABO blood group phenotypes and allele frequencies with CHIK infection.

## 1. Introduction

Chikungunya (CHIK) is a disease caused by arthropod-borne viruses transmitted by* Aedes* mosquitoes. Classically, acute infection manifests as the sudden onset of high-grade fever, rash, and severe joint pain [1]. The outbreak in Thailand during 2008–2010 showed that the highest attack rate, 1130.67 per 100,000 of population, was in Southern Thailand [2]. Compared with other arboviral diseases such as dengue disease, relatively few studies have been conducted in the host factors for CHIK infection.

ABO and Rhesus (Rh) blood groups are the most clinically important in transfusion practice and have been widely researched in population genetics, anthropological studies, and disease susceptibility studies [3–5]. Associations between the ABO blood group and various viral infections have been demonstrated, including dengue [6] and hepatitis C [7]. Currently, results from genome-wide association studies (GWAS) suggest an association of the ABO blood group antigen with systemic inflammation [8, 9].

Although the ABO blood group has been shown to play an important role in resistance or susceptibility to infections [10, 11], well-designed studies aimed at defining the relationship of the ABO blood group phenotype or allele frequency and susceptibility to CHIK infection are limited. Therefore, this study was undertaken to investigate the association of the ABO and Rh blood group phenotype, as well as allele frequency, with anti-CHIK IgG seropositivity in a community setting.

## 2. Material and Methods

We conducted this study in three villages in Phatthalung province in Southern Thailand because they had the highest reported CHIK infection rate, 45.89 per 100,000 of population, in 2010. Thai villagers aged ≥18 years, living in the study area during the CHIK outbreak, were enrolled. Exclusion criteria were as follows: having laboratory confirmation of other infections, congenital or acquired immune deficiencies, or a history of chronic small joint diseases. During August and September 2010, 506 subjects were enrolled. This study was approved by the Ethics Committee of Prince of Songkla University (EC 53-317-05-1-3), and written informed consent was obtained from all the participants.

### 2.1. ABO, Rh, and Anti-CHIK Serological Testing

We collected EDTA blood and tested blood groups at the Blood Bank, Songklanagarind Hospital. Within six hours after collection, both the ABO and Rh blood groups were determined with cell grouping using anti-A, anti-B, and anti-D antibodies (National Blood Centre, Thai Red Cross Society, Bangkok, Thailand). For the ABO blood group, we also performed serum grouping using A-cell and B-cell (National Blood Centre, Thai Red Cross Society, Bangkok, Thailand). Plasma was kept at −70°C before sending samples to the Armed Forces Research Institute of Medical Science (AFRIMS), Bangkok, for Anti-CHIK IgG hemagglutination inhibition (HI) test. We defined cases of CHIK infection as participants who had HI titre ≥1 : 10 [12, 13].

### 2.2. Statistical Analysis

Analyses were performed using R software with Epicalc packages. The blood group frequency was tested with chi-square or Fisher's exact tests, and odds ratios (ORs) with 95% confidence intervals (CI) were calculated. Symptomatic infection was defined as individuals with anti-CHIK IgG ≥ 1 : 10 who reported having acute fever with pain at the small joints during the CHIK outbreak. For symptomatic variables with three-category outcomes, including noninfected, symptomatic, and asymptomatic groups, we used polytomous logistic regression to calculate ORs and 95% CI.

To estimate the allele frequencies of the ABO blood group, we applied the Bernstein method as previously described [14, 15]. Briefly, the allelic frequencies of alleles A, B, and O were assigned as *p*, *q*, and *r*, respectively. Then, *p* = 1 − square root of [frequency (B) + frequency (O)], *q* = 1 − square root of [frequency (A) + frequency (O)], and *r* = 1 − square root of frequency (*O*). If *p* + *q* + *r* was not equal to 1, a deviation (*D*) = 1 − (*p* + *q* + *r*) was used to adjust the allelic frequencies as follows: *p*′ = *p*(1 + *D*/2), *q*′ = *q*(1 + *D*/2), and *r*′ = (*r* + *D*/2)(1 + *D*/2). The Hardy-Weinberg equilibrium (HWE) was tested using the goodness-of-fit chi-square test. The calculation for allelic frequencies and HWE was done using S2 ABOestimator software version 1.1.0.2 (Pedro J.N., Silva, Lisbon, Portugal). All *P* values less than 0.05 were considered significant.

## 3. Results

From a sample size of 506, we found 314 laboratory-confirmed CHIK cases (62.1%). Of the 314 cases, 166 had symptomatic infection. Median ages (IQR) of cases and controls were 47 (39, 58) and 45 (35.5, 58.5) years, respectively (*P* = 0.20).

All tested samples were Rh positive. No ABO discrepancy between cell and serum grouping was found. Distribution of positive and negative anti-CHIK IgG was significantly different in blood group O, A, and B (Table 1). The 95% CI of OR for blood groups A, B, and AB compared with O included unity, as shown in Table 1. The chi-square test between the ABO blood group and CHIK seropositivity showed no statistical significance (*P* value = 0.76).

For symptomatic manifestation with noninfection as a reference, the odds of being asymptomatic increased by 1.8-fold in age group of 40–60 years and decreased by 0.6 times in females (Table 2).

Allelic frequencies of the ABO blood group in each group, including CHIK seronegativity, seropositivity, and asymptomatic and symptomatic infection, are displayed in Table 3. There was no significant difference between the ABO allele frequencies and each outcome compared with CHIK seronegativity.

## 4. Discussion

This study demonstrated a very high rate of CHIK seropositivity confirmed by anti-CHIK IgG in a community-based study after an outbreak in Thailand. We also provided serological and allelic frequencies of the ABO blood group in Thais. However, the results did not show any significant association of ABO phenotype or allele frequencies with CHIK seropositivity and asymptomatic or symptomatic infection using CHIK seronegativity as a reference.

The ABO blood group distribution in this study was similar to other studies carried out in blood donors in Thailand [16, 17]. During an Indian outbreak in 2005–2009, two studies were conducted to investigate the relationship between ABO/Rh blood groups and CHIK infection based on self-declared symptoms. The results showed an increased susceptibility to CHIK infection in the Rh positive individuals [18]. The differences of the ABO blood group system between infected and noninfected groups were observed when combined with Rh status [19]. Generally, complex diseases especially host susceptibility to infection are influenced by more than one genetic or environmental factor. Therefore, extensive genetic studies are required to answer this question.

The limitation of the present study is that there was no anti-CHIK IgM or convalescent samples of IgG tested for confirming acute CHIK infection because we conducted this study one year after the outbreak. However, Panning et al. [20] reported no evidence of CHIK antibodies before an outbreak in Sri Lanka, where the last severe CHIK epidemic occurred during the same period in Thailand in 1960s [21].

In conclusion, our study demonstrated no association of the ABO blood group phenotype and allele frequencies with CHIK seropositivity. Further research should be undertaken in order to extensively explore the host and environmental factors associated with susceptibility and resistance to CHIK infection. This knowledge will help to identify susceptible individuals for monitoring and may be applied to other serious infections as well.

## Figures and Tables

**Table 1:** Univariate analysis for CHIK seropositivity.

Variables	Anti-CHIK IgG		*P* value	OR (95% CI)
	Positive (%) *N* = 314	Negative (%) *N* = 192		
Age (years)			0.13	
<40	97 (30.9)	76 (39.6)		Reference
40–60	148 (47.1)	80 (41.7)		1.45 (0.97, 2.17)
>60	69 (22)	36 (18.8)		1.5 (0.91, 2.48)
Gender			**0.03**	
Male	110 (35)	49 (25.5)		Reference
Female	204 (65)	143 (74.5)		0.64 (0.43, 0.95)
ABO blood group			0.76	
O	130 (41.4)	78 (40.6)	<0.001	Reference
A	80 (25.5)	46 (24)	0.003	1.04 (0.66, 1.65)
B	80 (25.5)	48 (25)	0.005	1 (0.63, 1.58)
AB	24 (7.6)	20 (10.4)	0.55	0.72 (0.37, 1.39)

**Table 2:** Univariate analysis for symptomatic manifestations.

Variables	Asymptomatic		Symptomatic	
	RRR	95% CI	RRR	95% CI
Age (years)				
<40	Reference		Reference	
40–60	**1.8**	**1.09, 2.97**	1.34	0.84, 2.13
>60	1.79	1, 3.21	0.82	0.45, 1.49
Gender				
Male	Reference		Reference	
Female	**0.6**	**0.38, 0.95**	0.66	0.42, 1.05
ABO blood group				
O	Reference		Reference	
A	1.05	0.65, 1.8	1.07	0.63, 1.82
B	1.13	0.67, 1.92	0.91	0.53, 1.55
AB	0.56	0.24, 1.31	0.86	0.41, 1.78

RRR: relative risk ratio.

**Table 3:** Allelic frequencies of ABO blood group in each group.

Group	Allelic frequency			HWE test *P* value
	*p*(A)	*q*(B)	*r*(O)	
CHIK seronegativity	0.19	0.19	0.62	0.06
CHIK seropositivity	0.18	0.18	0.64	0.38
(i) Asymptomatic infection	0.17	0.19	0.64	0.71
(ii) Symptomatic infection	0.19	0.17	0.63	0.12

HWE: the Hardy-Weinberg equilibrium.

## References

[1] Borgherini G., Poubeau P., Staikowsky F. (2007). Outbreak of chikungunya on Reunion Island: early clinical and laboratory features in 157 adult patients. *Clinical Infectious Diseases*.

[2] Bureau of Epidemiology (2010). *Situation of Chikungunya Fever in Thailand*.

[3] Ségurel L., Gao Z., Przeworski M. (2013). Ancestry runs deeper than blood: the evolutionary history of ABO points to cryptic variation of functional importance. *BioEssays*.

[4] Lee B. K., Zhang Z., Wikman A., Lindqvist P. G., Reilly M. (2012). ABO and RhD blood groups and gestational hypertensive disorders: a population-based cohort study. *British Journal of Obstetrics and Gynaecology*.

[5] Pelzer U., Klein F., Bahra M. (2013). Blood group determinates incidence for pancreatic cancer in Germany. *Frontiers in Physiology*.

[6] Kalayanarooj S., Gibbons R. V., Vaughn D. (2007). Blood group AB is associated with increased risk for severe dengue disease in secondary infections. *The Journal of Infectious Diseases*.

[7] Behal R., Jain R., Behal K. K., Dhole T. N. (2010). Variation in the host ABO blood group may be associated with susceptibility to hepatitis C virus infection. *Epidemiology and Infection*.

[8] Paré G., Chasman D. I., Kellogg M. (2008). Novel association of ABO histo-blood group antigen with soluble ICAM-1: results of a genome-wide association study of 6,578 women. *PLoS Genetics*.

[9] Melzer D., Perry J. R. B., Hernandez D. (2008). A genome-wide association study identifies protein quantitative trait loci (pQTLs). *PLoS Genetics*.

[10] Greenwell P. (1997). Blood group antigens: molecules seeking a function?. *Glycoconjugate Journal*.

[11] Anstee D. J. (2010). The relationship between blood groups and disease. *Blood*.

[12] Hammon W. M., Sather G. E. (1964). Virological findings in the 1960 hemorrhagic fever epidemic (dengue) in Thailand. *The American Journal of Tropical Medicine and Hygiene*.

[13] Theamboonlers A., Rianthavorn P., Praianantathavorn K., Wuttirattanakowit N., Poovorawan Y. (2009). Clinical and molecular characterization of Chikungunya virus in south Thailand. *Japanese Journal of Infectious Diseases*.

[14] Nam J. M., Gart J. J. (1976). Bernstein's and gene-counting methods in generalized ABO-like systems. *Annals of Human Genetics*.

[15] Ndoula S. T., Noubiap J. J. N., Nansseu J. R. N., Wonkam A. (2014). Phenotypic and allelic distribution of the ABO and Rhesus (D) blood groups in the Cameroonian population. *International Journal of Immunogenetics*.

[16] Nathalang O., Kuvanont S., Punyaprasiddhi P., Tasaniyanonda C., Sriphaisal T. (2001). A preliminary study of the distribution of blood group systems in Thai blood donors determined by the gel test. *The Southeast Asian Journal of Tropical Medicine and Public Health*.

[17] Chandanayingyong D., Sasaki T. T., Greenwalt T. J. (1967). Blood groups of the Thais. *Transfusion*.

[18] Kumar N. C., Nadimpalli M., Vardhan V. R., Gopal S. D. (2010). Association of ABO blood groups with Chikungunya virus. *Virology Journal*.

[19] Lokireddy S., Sarojamma V., Ramakrishna V. (2009). Genetic predisposition to chikungunya—a blood group study in chikungunya affected families. *Virology Journal*.

[20] Panning M., Wichmann D., Grywna K. (2009). No evidence of chikungunya virus and antibodies shortly before the outbreak on Sri Lanka. *Medical Microbiology and Immunology*.

[21] Thavara U., Tawatsin A., Pengsakul T. (2009). Outbreak of chikungunya fever in Thailand and virus detection in field population of vector mosquitoes, *Aedes aegypti* (L.) and *Aedes albopictus* Skuse (Diptera: Culicidae). *The Southeast Asian Journal of Tropical Medicine and Public Health*.

